# Peripheral nerve regeneration using a biodegradable conduit from açaí (*Euterpe oleracea*): a bio-based alternative to autografts

**DOI:** 10.1590/acb407725

**Published:** 2025-11-10

**Authors:** Rui Sergio Monteiro de Barros, Deivid Ramos dos Santos, Vitor Nagai Yamaki, Renan Kleber Costa Teixeira, André Lopes Valente, Tiago Santos Silveira, Carmen Gilda Barroso Tavares Dias

**Affiliations:** 1 Hospital Porto Dias – Departamento de Ortopedia e Traumatologia – Belém (PA) – Brazil.; 2 Hospital Porto Dias – Departamento de Ortopedia e Traumatologia – Programa de Pós-Graduação em Cirurgia Experimental – Belém (PA) – Brazil.; 3 Universidade de São Paulo – Faculdade de Medicina – Departamento de Neurocirurgia – São Paulo (SP) – Brazil.; 4 Universidade do Estado do Pará – Programa de Pós-Graduação em Cirurgia e Pesquisa Experimental – Belém (PA) – Brazil.; 5 Universidade do Estado do Pará – Faculdade de Medicina – Departamento de Neurologia – Belém (PA) – Brazil.; 6 Universidade Federal do Pará – Faculdade de Engenharia Química – Departamento de Engenharia de Materiais – Belém (PA) – Brazil.

**Keywords:** Peripheral Nerve Injuries, Autografts, Models, Animal

## Abstract

**Purpose:**

To evaluate the regenerative efficacy of an experimental biodegradable nerve conduit composed of polycaprolactone and açaí-derived polyurethane, used for peripheral nerve repair, in comparison with different reconstructive techniques.

**Methods:**

Wistar rats (n = 48) were allocated into six groups (n = 8): normality (NG), denervated (DG), burial (BG), nerve autograft (NAG), açaí-based neurotube (ANG), and vein autograft (VAG). Sciatic functional index, electrophysiological parameters, and histomorphometry were assessed after 12 weeks.

**Results:**

NAG and ANG showed significant functional recovery, with ANG being the only group to demonstrate progressive improvement (*p* = 0.009). Electrophysiological analysis revealed higher amplitude and lower latency in NAG, followed by ANG and VAG. Histomorphometric analysis showed increased axonal density in ANG and NAG compared to non-reconstructed groups (*p* = 0.004 and *p* = 0.007, respectively).

**Conclusion:**

The polycaprolactone/açaí-based conduit demonstrated regenerative performance comparable to autologous nerve grafts.

## Introduction

Peripheral nerve injuries showed a considerable clinical challenge, frequently resulting in functional deficits and diminished quality of life^1^. While peripheral nerves possess an inherent capacity for regeneration, this process is constrained in cases of extensive gaps or intricate lesions^2^. Autologous nerve grafting is still considered the gold standard for bridging these defects^3^^,^ 4. Despite being the current gold standard, this technique is associated with several complications, including the potential for donor site morbidity, neuroma formation, sensory loss, and limited graft availability^5^^–^ 7.

To address these limitations, biological and synthetic nerve conduits have been proposed as alternatives^8^. The utilization of conduits offers several advantages, including the avoidance of complications arising from the donor site and the capacity for structural customization^9^. Several biomaterials, including collagen-based tubes, chitosan scaffolds, and polycaprolactone conduits, have demonstrated efficacy in repairing peripheral nerve gaps of up to 3 cm in preclinical and limited clinical studies, although results in larger gaps remain suboptimal without adjuncts such as growth factors or stem cells^10^^,^ 11. Consequently, the quest for conduits that are biocompatible, porous, flexible, and biodegradable remains a focal point in nerve regeneration research^12^.

Recent advances in biomaterials research have identified natural sources, such as the Amazonian açaí (*Euterpe oleracea* Mart.), as promising candidates for biomedical applications. Polymeric derivatives obtained from its seeds have shown potential for developing biodegradable scaffolds with adequate porosity, mechanical stability, and structural integrity, making them suitable for supporting axonal growth and peripheral nerve repair^13^. Based on these properties, this study aimed to evaluate the feasibility and effectiveness of an açaí-derived bioconduit as a bio-based alternative to autologous nerve grafts in peripheral nerve injury repair.

## Methods

The study followed Brazilian legislation on animal experimentation (Law no. 11,794/08), based on National Institutes of Health guidelines and in compliance with the Council for International Organizations of Medical Sciences ethical code and ARRIVE guidelines. The protocol (approval number 16/2024) was approved by the Animal Use and Care Committee of the Universidade do Estado do Pará.

Forty-eight female Wistar rats (3 months, 200–300 g) were housed under controlled conditions. Room temperature was maintained at 22 ± 2°C, relative humidity at 50–60%, and animals were exposed to a 12-hour light/dark cycle (lights on at 7 a.m.) with controlled noise levels at the Experimental Surgery Laboratory, with food and water provided *ad libitum*. Animals were kept in non-sterile polyurethane cages (three or four per cage) with sterile wood shavings and no environmental enrichment.

**Figure 1:**
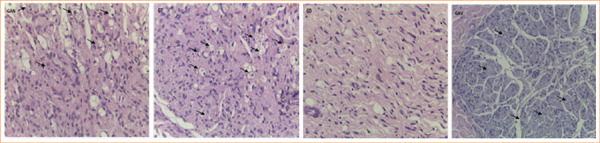
Implantation of the biodegradable neurotube in the sciatic nerve after a 10-mm gap and neurorrhaphy. The conduit was positioned between the proximal and distal nerve stumps and sutured with 10-0 nylon.

Source: Elaborated by the authors.

**Figure 2:**
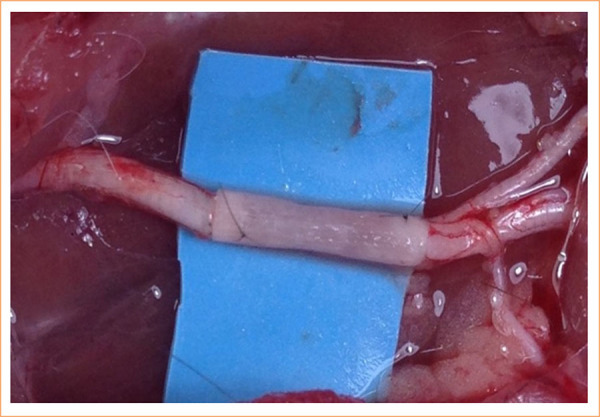
Surgical procedure of autologous vein graft interposition in the sciatic nerve. The external jugular vein was harvested and used to bridge a 10-mm nerve gap, fixed with 10-0 nylon epineural sutures.

Source: Elaborated by the authors.

The animals were randomly allocated into six groups (n = 8/group). Each rat was assigned a numerical code, and group allocation was determined through a computer-generated random sequence created in BioEstat version 5.4, ensuring unbiased distribution among experimental groups:

- Normality group (NG): simulated surgery without nerve injury;
- Denervated group (DG): the sciatic nerve was surgically exposed and sectioned in its middle portion, resulting in a 10-mm nerve gap without reconstruction;
- Burial group (BG): the proximal nerve stump was buried in adjacent muscle tissue after transection;
- Nerve autograft group (NAG): a 10-mm segment of the sciatic nerve was resected and immediately reimplanted in its original anatomical orientation using two simple 10-0 nylon epineural sutures at each stump;
- Açaí-based neurotube group (ANG): a 10-mm nerve gap was created and bridged using a 13-mm biodegradable neurotube composed of açaí-based polymer. The conduit was interposed between the proximal and distal stumps and fixed with two 10-0 nylon epineural sutures per side (Fig. 1);
- Vein autograft group (VAG): similarly, after the creation of a 10-mm nerve gap, an 11-mm segment of autologous external jugular vein was harvested through a lateral cervical incision, ligated at both ends, and interposed between the stumps. The vein graft was secured with two 10-0 nylon epineural sutures at each end (Fig. 2).

### Fabrication of the nerve conduit

The nerve conduit was fabricated from a blend of polycaprolactone (PCL) and bio-based polyurethane (PU) derived from *E. oleracea* Mart. (açaí) seed polyol, selected for their biocompatibility, biodegradability, and mechanical suitability. PCL was solubilized in dichloromethane and combined with PU synthesized via a two-step reaction using hexamethylene diisocyanate. The mixture underwent rotary spinning at 3,450 rpm, generating fibrous, porous structures wrapped around 0.6-mm catheters to form 2–3 cm tubular scaffolds. Porosity (60–80%) and pore size (20–30 µm) were controlled to mimic the extracellular matrix and promote axonal growth and vascularization.

#### Physicochemical and mechanical characterization

The conduit was characterized for water absorption, density, porosity, and compressive strength. Water uptake was calculated by comparing sample masses before and after 24-hour immersion in distilled water at 22°C. Apparent density was determined using a calibrated pycnometer. Porosity was quantified via ethanol displacement. Mechanical properties were assessed by compressive testing (ASTM D695-96), applying 40% deformation to 6 × 3-mm cylindrical specimens. The addition of PU enhanced flexibility and mechanical resilience, supporting structural integrity during nerve regeneration.

All surgical procedures were carried out by a single microsurgeon with over 25 years of experience, utilizing a magnified video system^14^. Anesthesia was induced with intraperitoneal ketamine (70 mg/kg) and xylazine (10 mg/kg)^15^. After antisepsis of the right thigh, a posterolateral longitudinal incision was made, exposing the sciatic nerve and its branches (common fibular, tibial, sural) at the popliteal fossa.

In all animals, the aponeurotic layer was sutured with 5-0 nylon sutures, and the skin with continuous 6-0 nylon sutures. Lactated Ringer’s solution (5 mL/kg intravenously, via the lateral tail vein using a 26-gauge catheter) was administered postoperatively. No antibiotics or anticoagulants were used. The duration of the surgical procedure was meticulously documented.

Motor function was assessed at four, eight, and 12 weeks postoperatively using the sciatic functional index, based on hind paw print analysis after inkpad walking across an 8.7 × 43-cm track^16^.

Electrophysiological recordings of the tibial and common peroneal nerves were obtained at 12 weeks post-surgery under anesthesia, in accordance with the standardized protocol of Somensi et al.^17^.

**Figure 3:**
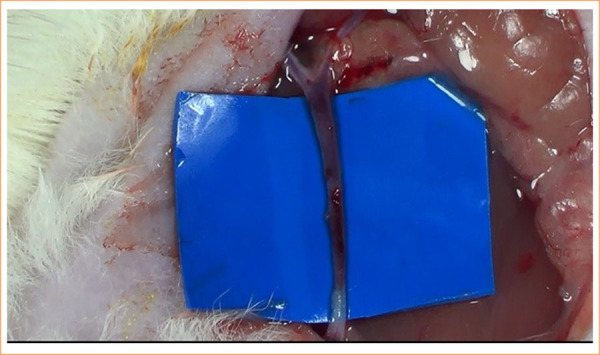
Histological analysis of distal sciatic nerve regeneration across experimental groups. Hematoxylin and eosin staining, 400x magnification. Black arrows indicate axons surrounded by Schwann cells.

Source: Elaborated by the authors.

After electroneuromyography, animals were euthanized via an intraperitoneal overdose of ketamine (500 mg/kg) combined with xylazine (300 mg/kg), in accordance with institutional guidelines and international recommendations for animal research ethics. The right gastrocnemius muscle was carefully dissected and immediately weighed using a calibrated precision scale. The wet muscle mass was recorded in milligrams. Distal segments of the sciatic nerve were fixed in 10% buffered formalin for 24 hours, dehydrated in graded ethanol, cleared in xylene, sectioned at 4 µm, and stained with hematoxylin and eosin. Axonal density was analyzed in 10 randomly selected fields at 400× magnification under light microscopy (Fig. 3).

Statistical analysis was performed using BioEstat 5. Data normality was assessed with the Shapiro-Wilk test, followed by one-way analysis of variance (ANOVA) for group comparisons. A significance level of *p* < 0.05 was adopted. A sample size of eight animals per group was defined based on an estimated effect size (f = 1.33), considering a 20-point difference in SFI, standard deviation of 15, α = 0.05, and power > 80%. This ensured sufficient sensitivity for detecting intergroup differences.

## Results

All animals completed the experimental protocol without incident or need for replacement, and no mortality was observed.

Baseline analysis confirmed group homogeneity in body weight (*p* = 0.0064), ensuring balanced distribution. Muscle mass was significantly greater in the NG group (0.57 ± 0.12 g; *p* < 0.001), whereas DG (0.14 ± 0.10 g) and BG (0.16 ± 0.03 g) showed marked muscle atrophy. Reconstruction groups—NAG, ANG, and VAG—demonstrated partial preservation of muscle trophism, with no significant differences among them (Table 1).

**Table 1:** Descriptive analysis of body weight and gastrocnemius muscle preservation across groups * .

Group	Body weight (g)	Min	Max	Gastrocnemius Mass(g)	Min	Max
NG	231.7 ± 19.69	195	255	0.57 ± 0.12	0.45	0.61
DG	202.5 ± 26.20	169	245	0.14 ± 0.10	0.10	0.18
BG	210 ± 23.19	175	246	0.16 ± 0.03	0.11	0.20
NAG	229.5 ± 26.47	185	271	0.25 ± 0.04	0.13	0.29
ANG	205.8 ± 23.81	189	235	0.24 ± 0.08	0.17	0.39
VAG	215.0 ± 21.0	180	240	0.20 ± 0.05	0.12	0.25

* Analysis of variance (ANOVA) (body weight): *p* = 0.0064;

ANOVA (muscle mass): *p* < 0.001; NG: normality group; DG: denervated group; BG: burial group; NAG: nerve autograft group; ANG: açaí-based neurotube group; VAG: vein autograft group. Source: Elaborated by the authors.

### Functional recovery: sciatic nerve functional index

SFI analysis revealed marked functional deficits in the early postoperative period, particularly in BG (-81.54 ± 9.55) and DG (-75.76 ± 8.32), both significantly lower than NG (-18.60 ± 7.76). The NAG group showed progressive improvement, reaching -66.34 ± 12.74 by the third month. Notably, ANG exhibited significant functional recovery between the first and third months (*p* = 0.009), indicating continuous regenerative activity. VAG demonstrated modest improvement (-70.04 ± 10.22 at month 3), but this difference was not statistically significant compared with DG or BG (*p* = 0.057), suggesting only a trend toward inferior functional outcomes relative to other reconstruction strategies (Table 2).

**Table 2:** Sciatic Functional Index progression over three months in experimental groups.

Group	1st Month	2nd Month	3rd Month	*p*-value
NG	-18.60 ± 7.76	17.90 ± 7.5	17.80 ± 7.8	*p* = 0.083
DG	-75.76 ± 8.32	-75.21 ± 7.27	-73.22 ± 5.01	*p* = 0.089
BG	-81.54 ± 9.55	-81.04 ± 7.98	-80.06 ± 8.81	*p* = 0.077
NAG	-65.50 ± 8.21	-68.15 ± 8.19	-66.34 ± 12.74	*p* = 0.066
ANG	-73 ± 3.80	-66.89 ± 3.38	-59.15 ± 12.38	*p* = 0.009
VAG	-79.75 ± 9.77	-73.04 ± 16.15	-70.04 ± 10.22	*p* = 0.057

NG: normality group; DG: denervated group; BG: burial group; NAG: nerve autograft group; ANG: açaí-based neurotube group; VAG: vein autograft group. Source: Elaborated by the authors.

Statistical analysis (paired t-test for intragroup comparisons and ANOVA with Tukey’s post-hoc test) showed that only the ANG group demonstrated statistically significant improvement between the first and third postoperative months (*p* = 0.009). Intragroup comparisons for DG (*p* = 0.089), BG (*p* = 0.077), NAG (*p* = 0.066), and VAG (*p* = 0.057) did not reach statistical significance. At the second and third months, NAG, DG, and VAG were statistically similar, whereas ANG was the only group with significantly better scores than DG and BG (*p* = 0.01). No differences were found between DG and BG (*p* > 0.05).

### Electrophysiological analysis

Electrical conduction through the common peroneal nerve was detected only in the reconstructed groups (Tables 3 and 4). The NAG group showed the lowest latency (1.57 ± 0.28 ms) and the highest amplitude (11.08 ± 8.79 mV), with statistically significant differences compared to ANG (2.35 ± 1.06 ms; 7.91 ± 5.52 mV, *p* = 0.010) and VAG (2.39 ± 0.73 ms; 5.68 ± 2.94 mV, *p* = 0.018). No significant differences were observed between ANG and VAG.

**Table 3:** Electrophysiological latency (ms) of the common peroneal and tibial nerves across experimental groups * .

Group	Common peroneal nerve	Min	Max	*p* -value	Tibial nerve	Min	Max	*p* -value
NG	1.15 ± 0.09	1.0	1.3	*p* = 0.001	1.30 ± 0.15	1.1	1.5	*p* = 0.010
DG	0 ± 0	0	0	-	0 ± 0	0	0	-
BG	0 ± 0	0	0	-	0 ± 0	0	0	-
NAG	1.57 ± 0.28	1.2	2.0	*p* = 0.01	1.35 ± 0.22	1.0	1.6	*p* = 0.001
ANG	2.35 ± 1.06	1.9	3.9	*p* = 0.043	1.42 ± 0.43	1.9	3.9	*p* = 0.010
VAG	2.39 ± 0.73	1.3	3.65	*p* = 0.036	2.18 ± 0.86	1	3.35	*p* = 0.018

NG: normality group; DG: denervated group; BG: burial group; NAG: nerve autograft group; ANG: açaí-based neurotube group; VAG: vein autograft group;

* statistical analysis: one-way analysis of variance was performed separately for each nerve, followed by Tukey’s post-hoc test for multiple comparisons;

Common peroneal nerve: NAG exhibited significantly lower latency than both ANG and VAG, while no difference was observed between ANG and VAG; Tibial nerve: NAG and ANG showed significantly better latency results than VAG, while no difference was observed between ANG and VAG. ource: Elaborated by the authors.

**Table 4:** Amplitude of stimulus response in the common peroneal and tibial nerves of experimental groups (mV).

Group	Common peroneal nerve	Min	Max	*p* -value	Tibial nerve	Min	Max	*p*-value
NG	30.98 ± 5.55	26.7	40.2	*p* < 0.001	39.88 ± 8.85	30.8	52.2	*p* < 0.001
DG	0 ± 0	0	0	-	0 ± 0	0	0	-
BG	0 ± 0	0	0	-	0 ± 0	0	0	-
NAG*	11.08 ± 8.79	1.5	17.8	*p* = 0.018	15.35 ± 8.50	9.7	25.9	*p* = 0.027
ANG*	7.91 ± 5.52	1.9	3.9	*p* = 0.021	10.24 ± 9.21	2.0	20.1	*p* = 0.034
VAG	5.68 ± 2.94	2.9	10.8	*p* = 0.01	3.93 ± 2.41	1.1	7.8	*p* = 0.041

NG: normality group; DG: denervated group; BG: burial group; NAG: nerve autograft group; ANG: açaí-based neurotube group; VAG: vein autograft group. Source: Elaborated by the authors.

The NAG group demonstrated superior performance (latency = 1.35 ± 0.22 ms; amplitude = 15.35 ± 8.50 mV) compared to VAG (2.18 ± 0.86 ms; 3.93 ± 2.41 mV) and ANG (1.42 ± 0.43 ms; 10.24 ± 9.21 mV), with *p* < 0.01. However, no significant differences were found between NAG and the other reconstructive groups in pairwise comparisons.

No measurable electrical activity was recorded in the DG and BG groups for either nerve.

### Amplitude: one-way analysis of variance with Tukey’s post-hoc test

Regarding the common peroneal nerve, NG showed significantly higher amplitude than all other groups. NAG and ANG showed higher amplitudes compared to VAG and BG. No significant differences were detected among reconstructed groups (NAG *versus* ANG, *p* = 0.412; ANG versus VAG, *p* = 0.354; NAG *versus* VAG, *p* = 0.278).

About the tibial nerve, NG differed significantly from all other groups. NAG and ANG showed higher amplitudes compared to VAG and BG (*p* = 0.027 and *p* = 0.034, respectively). NAG showed higher amplitude than VAG (*p* = 0.041), while no significant differences were observed between ANG and VAG (*p* = 0.296) or between ANG and NAG (*p* = 0.237).

Histomorphometric analysis showed significantly higher axonal density in the NG group (53.2 ± 7.1; *p* < 0.001). In contrast, DG (8.00 ± 2.1) and BG (7.11 ± 2.1) exhibited markedly reduced axon counts, consistent with regenerative failure (Table 5). Reconstruction groups—NAG (28.1 ± 7.0), ANG (22.2 ± 6.2), and VAG (18.1 ± 5.3) demonstrated significantly higher axonal density than non-reconstructed groups (*p* < 0.05), with no significant differences among themselves.

**Table 5:** Myelinated axon count in the distal sciatic nerve by experimental group.

Group	Mean ± SD (axons/field, 40×)	*p* -value
NG*	53.2 ± 7.1	*p* = 0.002
DG	8.00 ± 2.1	*p* = 0.611
BG	7.11 ± 2.1	*p* = 0.694
NAG**	22.2 ± 6.2	*p* = 0.004
ANG**	28.1 ± 7.0	*p* = 0.007
VAG**	18.1 ± 5.3	*p* = 0.021

NG: normality group; DG: denervated group; BG: burial group; NAG: nerve autograft group; ANG: açaí-based neurotube group; VAG: vein autograft group; SD: standard deviation; statistical analysis: data distribution was assessed using the Shapiro-Wilk test, which indicated deviations from normality across groups. Given the robustness of analysis of variance (ANOVA) for balanced samples, a one-way ANOVA was applied, followed by Tukey’s post-hoc test for pairwise comparisons. NG presented significantly higher axonal density compared to all other groups (p = 0.002). Reconstruction groups (NAG, ANG, VAG) demonstrated significantly greater axonal densities than DG and BG (p = 0.004, 0.007, and 0.021, respectively). No statistically significant differences were observed among the reconstructed groups (NAG versus ANG: p = 0.214; NAG versus VAG: p = 0.119; ANG versus VAG: p = 0.347). DG and BG did not differ significantly from each other (p = 0.611; p = 0.694).

Source: Elaborated by the authors.

## Discussion

This study evaluated an açaí-based biodegradable neurotube and compared it to established methods. Peripheral nerve injuries accompanied by segmental loss pose a significant challenge, particularly when the primary suture is not viable^18^^–^ 20. Although autologous grafting is widely regarded as the gold standard, its inherent limitations have prompted the exploration of alternative solutions.

No mortality or morbidity was observed in any of the experimental groups during the evaluation period. As expected, the autograft groups did not exhibit compatibility-related issues, given that the graft tissue originated from the same organism. The açaí-based neurotube, being the only non-autologous material tested, did not induce systemic toxicity or overt adverse effects, and its biocompatibility was supported by functional, electrophysiological, and histological findings approaching those of autografts in this early-stage model.

Conducted previously, the extant studies^6^^,^ 21 ^–^ 23 have reported an absence of adverse systemic effects with polymer conduits, even in critical defects. This finding underscores the notion that the structure and manufacturing process directly influence the biological response to the implant^24^^–^ 26. In the case of the açaí-conduit, its porous and biomimetic architecture promotes tissue integration without inducing an exacerbated inflammatory response^18^^,^ 19.

However, it is important to note that the present study did not evaluate the in-vivo degradation profile of the conduit. Although the polymeric blend of polycaprolactone and açaí-based polyurethane was selected based on prior biocompatibility and mechanical testing, the timeline of scaffold resorption, nature of biodegradation byproducts, and their interaction with surrounding tissues remain unexplored. Future investigations should address these parameters to clarify the long-term performance and translational viability of the material in clinical scenarios. Consequently, the integration of bioactive materials with adapted manufacturing methodologies can yield an effective regenerative milieu without compromising animal welfare.

Regarding the preservation of muscle mass, while all the operated groups exhibited significant atrophy of the gastrocnemius relative to the NG, the ANG group showed better muscle preservation than DG and VG, and slightly more than AG, though not statistically significant. Previous studies have demonstrated that the integrity of muscle mass serves as a metric for the effectiveness of motor reinnervation, with factors such as the type of conduit, the time of assessment, the animal species, and the extent of nerve damage contributing to this effectiveness^12^^,^ 18 ^,^ 20.

Only the ANG group showed significant SFI improvement over time, indicating progressive axonal regeneration between the initial and third months, suggesting that the neurotube possessed the capacity to facilitate progressive axonal regeneration and enable functional reconnection with the motor plates. This recovery pattern is consistent with the findings observed in studies of chitosan and collagen-based conduits, which have demonstrated the capacity to enhance gait patterns and preserve locomotor functions in rats with critical sciatic nerve defects^21^. In addition, several studies have demonstrated that biomaterials with porous microarchitecture and bioactive properties not only favor axonal growth but also facilitate integration with adjacent muscle tissue, thereby reducing atrophy and functional deficit^20^^,^ 22 ^,^ 23.

Despite the prevalence and acceptance of venous grafts due to their biocompatibility, their effectiveness can be hindered when utilized without the inside-out technique. This technique exposes the adventitial layer, which is abundant in trophic factors and promotes the migration of Schwann cells^23^. In this study, the VG group exhibited suboptimal performance with respect to functional and morphological outcomes, a phenomenon that may be associated with the absence of this technical strategy.

Electrophysiological evaluation demonstrated that ANG and AG had superior electrophysiological responses than DG and VG, supporting effective reconduction that the açaí neurotube facilitated functional electrical reconduction. These findings underscore the notion that meticulously designed conduits can nearly reach the benchmark of autografts, particularly when exhibiting anisotropic organization, compatible mechanical strength, and cell support capacity^24^. Conductive polymer conduits, such as polycaprolactone, chitosan, and analogous biodegradable polymers, have demonstrated a progressive enhancement in electrophysiological parameters, even in lesions with substantial gaps, as reported in previous studies^25^.

Histomorphometric analysis revealed that AG and ANG exhibited significantly higher axon densities compared to DG and VG, with no statistical difference observed between the two groups. Although lower than the normal group, the number of regenerated axons in the ANG group was consistent with results observed in models with autologous grafts or conduits enriched with stem cells and growth factor^26^. These findings suggest that the Amazonian neurotube possesses the capacity to facilitate targeted axonal regeneration, even in the absence of additional biological enrichment.

Additionally, only female Wistar rats were used in this study. This choice was based on standardized protocols and prior institutional experience indicating greater procedural consistency and lower rates of territorial aggression among females, which improves reproducibility and animal welfare. Notably, previous studies have demonstrated that female rats may exhibit enhanced regenerative responses following peripheral nerve injury, including increased axonal elongation and accelerated sensory recovery^27^. Nonetheless, future investigations including both sexes are essential to assess potential sex-related biological differences.

The study presents two main methodological limitations. The 12-week follow-up period may have been insufficient to evaluate late regenerative events, such as functional myelination and neuromuscular reorganization. Furthermore, the absence of immunohistochemical analysis limits insights into cellular mechanisms such as Schwann cell migration and angiogenesis. Immunomarkers like NF-200 and S100 may enhance future analyses of the evaluation of axonal maturation and vascular support, representing a relevant focus for early-phase future investigations^28^.

These findings highlight the potential of the açaí-based neurotube as a bioengineered alternative for nerve repair. However, additional long-term and mechanistic studies are warranted to confirm its safety and clinical applicability.

## Conclusion

The açaí-based neurotube supports peripheral nerve regeneration, showing functional, electrophysiological, and histological outcomes comparable to autografts, without adverse effects.

## Data Availability

The data will be available upon request.
